# Genetic and molecular analysis of leaf blast resistance in Tetep derived line RIL4 and its relationship to genes at *Pita/Pita*^*2*^ locus

**DOI:** 10.1038/s41598-023-46070-7

**Published:** 2023-10-31

**Authors:** B. Biswas, K. Thakur, T. D. Pote, K. D. Sharma, S. Gopala Krishnan, A. K. Singh, T. R. Sharma, R. Rathour

**Affiliations:** 1 CSK Himachal Pradesh Agricultural University, Palampur, 176062 India; 2 College of Horticulture and Forestry, Dr YSP University of Horticulture and Forestry, Thunag, 175048 India; 3 Division of Genetics, Indian Agricultural Research Institute, New Delhi, 110012 India; 4 Indian Council of Agricultural Research, Krishi Bhawan, New Delhi, 110001 India

**Keywords:** Biotechnology, Genetics, Plant sciences

## Abstract

The Vietnamese *indica* landrace ‘Tetep’ is known worldwide for its durable and broad spectrum-resistance to blast. We performed genetic and molecular analyses of leaf blast resistance in a Tetep derived recombinant inbred line ‘RIL4’ which is resistant to both leaf and neck blast. Phenotypic analysis of segregating F_2_ progenies suggested that leaf blast resistance in RIL4 was controlled by a dominant gene tentatively designated as *Pi*-*l(t)*. The gene was mapped to a 2.4 cm region close to the centromere of chromosome 12. The search for the gene content in the equivalent genomic region of reference cv. Nipponbare revealed the presence of five NBS-LRR genes, two of which corresponded to the alleles of *Pita* and *Pi67* genes previously identified from Tetep. The two other genes, *LOC_Os12g17090, and LOC_Os12g17490* represented the homologs of stripe rust resistance gene *Yr10*. The allelic tests with *Pita2* and *Pi67* lines suggested that the leaf blast resistance gene in RIL4 is either allelic or tightly linked to these genes. The genomic position of the leaf blast resistance gene in RIL4 perfectly coincided with the genomic position of a neck blast resistance gene *Pb2* previously identified from this line suggesting that the same gene confers resistance to leaf and neck blast. The present results were discussed in juxtaposition with past studies on the genes of *Pita/Pita*^*2*^ resistance gene complex.

## Introduction

Rice [*Oryza sativa* (L.) 2*n* = 2*x* = 24] belongs to genus *Oryza* and family Poaceae. The genus is composed of 24 species, out of which two species *O. sativa* L. and *O. glaberrima* are cultivated^1^. Its high energy digestibility coupled with fact that it serves as the primary calorie source for nearly the one third of world’s population makes rice one of the most important cereal crops in the world^2,3^. During 2021 rice has been cultivated in an area of 165.25 million hectares in the world with annual production of 787.29 million tonnes^4^. With the current rate of population growth, the global rice demand is expected to escalate to 8.52 × 10^8^ tonnes by 2035 requiring further genetic improvements^5^. The yield gains achieved through plant breeding are frequently offset by the damage inflicted by various biotic and abiotic stresses allowing farmers to realize only half of the rice yield potential^6^. Among the biotic stresses, blast disease caused by hemibiotrophic ascomycete fungus *Pyricularia oryzae* Cavara (Telomorph*, Magnaporthe oryzae*), is one of the most widespread and destructive diseases. The disease results in yield loss of $66 billion worldwide annually, which is enough to feed 60 million people^7^. The disease affects crop at almost all growth stages leading to two easily recognizable phases of the disease: leaf blast occurring during the vegetative stage causing spindle shaped lesions on leaf blades and leaf collars, and neck blast (synonymous with panicle blast) which is characterized by infection at plant nodes and different parts of panicle and grains. The neck blast is economically more significant because a single infection at the panicle base can result in completely empty panicles, and losses up to 70% have been recorded in the crop affected by neck blast^8^. Despite the availability of many potent fungicides, potential environmental and health risks along with economic considerations make chemical control of the disease an ungainly prospect. Under such constraints, utilization of host resistance mediated by resistance genes is the most efficient practice to manage the disease. Identification of genes for resistance to leaf and neck blast and their utilization in breeding resistant varieties is the most efficient management practice to manage the disease.

Until now some 120 blast resistance genes have been identified and 25 of those have been cloned and molecularly characterized^9–14^. While a great many blast resistance genes identified till date are effective against leaf blast, only three, namely, *Pb1, Pi25* and *Pi64* are known to confer resistance to neck blast phase of the disease^15–17^. Reported susceptibility to one phase of disease in genotypes resistant to the other phase indicates that the mechanisms and underlying genetic factors involved in neck and leaf blast resistance may be different^8,18,19^. Under such circumstances the identification of genes that confer simultaneous resistance to both leaf and neck blast phases of the disease is urgently required for effective management of the disease.

The Vietnamese *indica* landrace ‘Tetep’ has shown remarkably broad spectrum and persistent resistance against various strains of the blast pathogen in different parts of the world^20,21^. The genotype has shown enduring resistance to both leaf and neck blast disease for several years in north-western Indian states of Himachal Pradesh, Uttarakhand and J&K^22^. Tetep has been widely adopted as the progenitor variety in several breeding programs due to its durable and broad spectrum resistance profile^23^. Till date six leaf blast resistance genes viz*., Pi-1, Pi-k*^*h*^ and *Pi54* on chromosome 11, and two putatively allelic genes *Pita* and *Pita2* and *Pi67* on chromosome 12 have been identified from the Tetep^24–29^. A new neck blast resistance gene, *Pb2* mapped to centromeric region of chromosome 12 has recently been identified from a recombinant inbred line RIL4 that derives its resistance from Tetep^30^. These studies suggested that the genomic region close to centromere of chromosome 12 of Tetep harbors a cluster of resistance genes that can exploited in rice breeding for developing cultivars resistant to both leaf and neck blast phase of the disease. The efficient exploitation of this region in resistance breeding, however, would require precise dissection of resistance spectrum of these genes to identify the genes that provide resistance to both leaf and neck blast phases the disease. In present study, we performed the genetic and molecular analyses of leaf blast resistance of RIL4, a recombinant inbred line derived from Tetep, that has previously been shown to harbor a neck blast resistance gene *Pb2* in order to clarify whether the same gene confers resistance to both leaf and neck blast or there are different genes for resistance to different phases of the disease. The allelic relationship of leaf blast resistance gene identified from RIL4 to leaf blast resistance genes *Pita2* and *Pi67* previously identified from the same region of Tetep was also investigated. The genetic mapping of leaf blast resistance gene from RIL4 will ensure effective and precise manipulation leaf and neck blast resistance gene(s) identified from this genotype in breeding blast resistant varieties.

## Results

### Genetic analysis of leaf blast resistance

**Figure 1:**
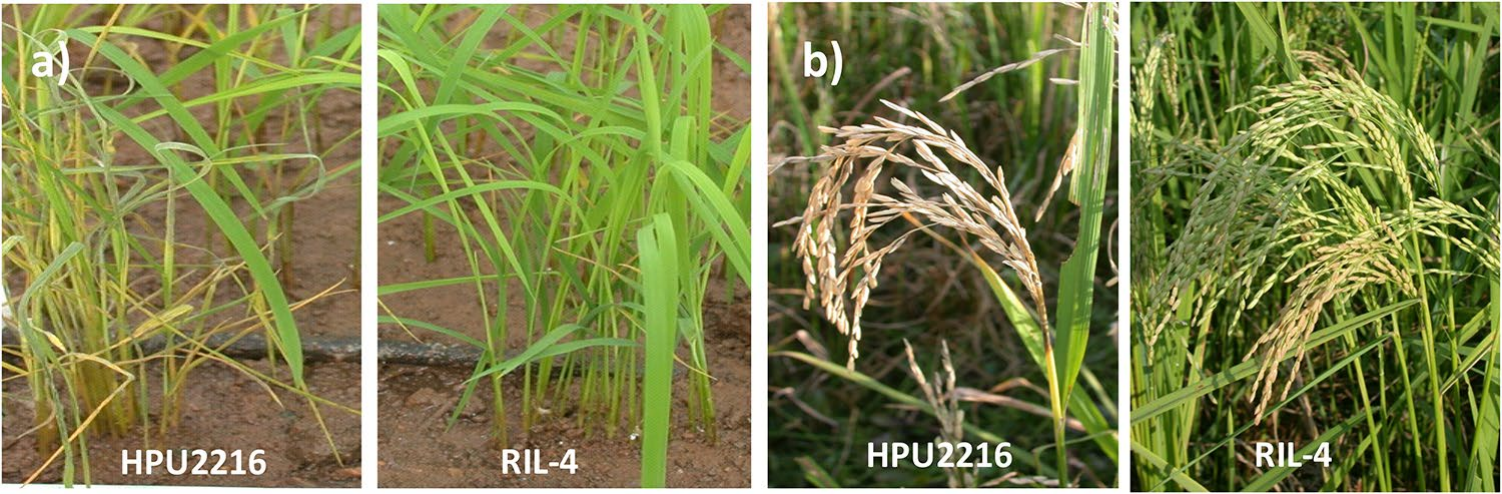
Disease reaction of parental genotypes HPU2216 and RIL4 to leaf and neck blast. (**a**) Reaction of parental genotypes to *Pyricularia oryzae* isolate *Po-HPU2216- 5–2* at seedling stage. (**b**) Reaction of parental genotypes to neck blast at grain filling stage.

Altogether nine hundred and thirty-four F_2_ plants derived from a cross between HPU2216 and blast resistant genotype RIL4 were inoculated with blast isolate *Po-HPU2216-5-2*. The genotype HPU2216 with *Pita* gene displayed susceptibility to leaf blast, while RIL4 was completely resistant showing no symptoms of the disease (reaction type 0) (Fig. 1). Of the total inoculated F_2_ seedlings, 683 exhibited resistant reaction, while 251 were susceptible to leaf blast. The segregation of resistant and susceptible plants showed a good fit to segregation ratio expected for a single dominant gene (Table 1). The new leaf blast resistance gene identified from RIL4 was tentatively designated as *Pi-l(t).*

**Table 1:** Segregation of resistance in an F_2_ population of a cross between leaf blast susceptible genotype HPU2216 and resistant genotype RIL4.

Cross	No. of plants (R:S)	Expected ratio (R: S)	χ^2^	*P* value
F_2_ (HPU 2216 × RIL4)	683:251	3 : 1	1.75	0.10 < *P* < 0.50

R = Resistant; S = susceptible. χ^2^ = The actual value of Chi-square test for resistant/susceptible ratio.

### Mapping of leaf blast resistance gene

**Figure 2:**
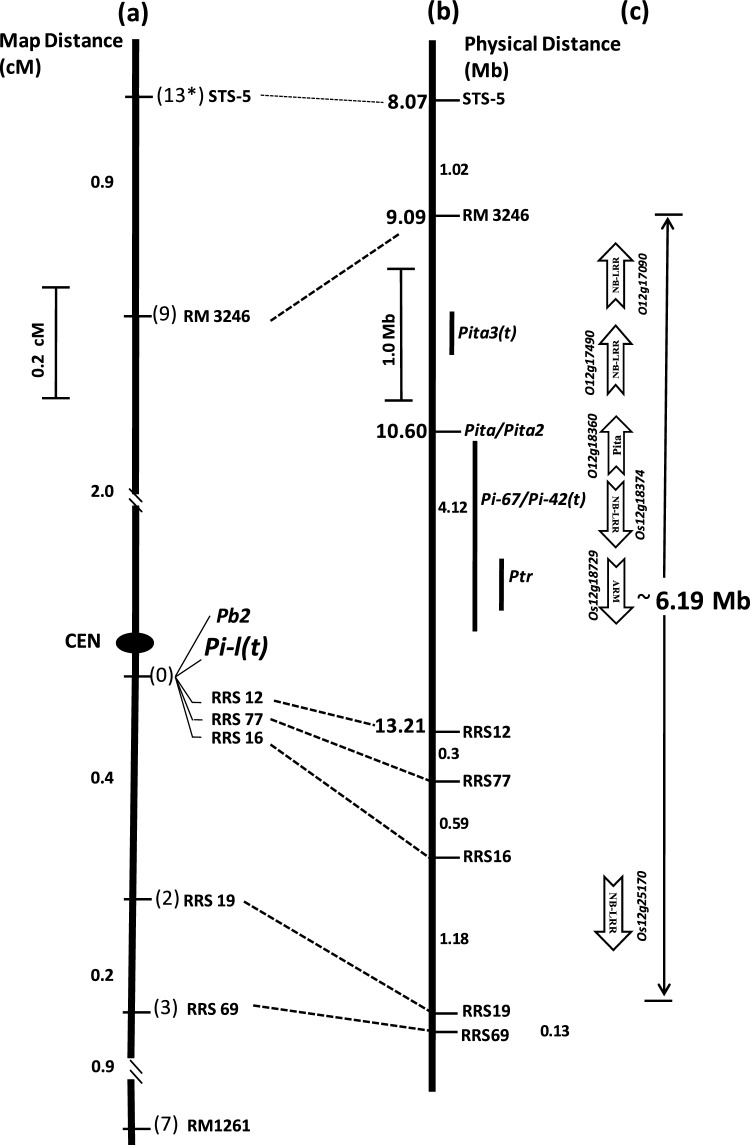
Genetic and physical map of leaf blast resistance gene identified from RIL4. (**a**) The genetic map of *Pi-l(t)* gene based on linkage analysis of F_2_ progeny of cross HPU2216 x RIL4. *The numbers of recombination events obtained between the relevant markers and *Pi-l(t)*. The numbers to the left of map are relative genetic distances in centimorgans. (**b**) The physical map of *Pi-l(t)* gene constructed by e-landing of closely linked markers on the genome sequence of cv. Nipponbare (IRGSP 1.0) released by International Rice Genome Sequencing Project (http://rapdb.dna.affrc.go.jp). The dotted lines designate the positions of the markers on the genome of cv. Nipponbare. The numbers to the right of the map are the distances between the markers in Mbp. The physical positions of *Pita3(t)* (9.88–10.06)*, Pita* (10.60)*, Pi67(t)* (10.61–12.09)*, Pi-42(t)* (8.07–12.24) and *Ptr* (10.71–10.98) in million base pairs (Mb) were are adapted from Chen et al.^31^, Jia and Martin^32^, Joshi et al.^29^, Kumar e al.^33^ and Meng et al.^34^. (**c**) Candidate resistance genes with NBS-LRR features identified in the region of *Pi-l(t)* by searching the TIGR Rice Genome Annotation Project database of cv. Nipponbare (http://rice.plantbiology.msu.edu). CEN: Centromere.

Since a single dominant gene *Pb2* located on chromosome 12 has previously been shown to confer neck blast resistance in line RIL4^30^, we initiated mapping of leaf blast resistance gene using the polymorphic markers from the same chromosome on premise that the leaf blast resistance may be conditioned by the similar gene. Initially, two polymorphic markers STS-5 and RM1261, one each from the short and long arm of chromosome 12, were tested for their linkage to leaf blast resistance gene in a mapping population comprising of 220 susceptible F_2_ plants of cross HPU2216 x RIL4. A total of 13 recombination events were detected between STS-5 and resistance gene, whereas 7 single cross events were detected with RM1261 in a population of 220 susceptible F_2_ plants (equivalent to 440 F_1_ gametes) thereby suggesting that the leaf blast resistance gene is also located on chromosome 12 (Supplementary Table S1; Supplementary Fig. S1). Since the recombinants detected with STS-5 and RM1261 were non-identical, these markers were inferred to flank the gene at a distance of 2.9 and 1.5 cm, respectively. Based on this data, the resistance gene was initially mapped to a 4.4 cm genetic interval flanked by STS-5 and RM1261 on chromosome 12 (Fig. 2). To further narrow down the genomic region for the resistance gene, chromosome walking was initiated with the markers located within the interval STS5-RM1261. Of the 11 recombinants detected with distal marker STS-5, 9 were detected by RM3246 marker, whereas of the 7 recombination events detected with RM1261, only three were detected at RRS69 locus (Fig. 2). Of the three recombinants detected with RRS 69, two were also detected with RRS19 (Fig. 2). With this analysis the genomic region for leaf blast resistance gene *Pi-l(t)* was eventually narrowed down to a 2.4 cm region defined by RM3246 located at 2.0 cm on telomeric side and RRS19 located at 0.4 cm from the gene on centromeric side. Within this narrow genetic interval three SSR markers viz., RRS12, RRS77 and RRS16 co-segregated with the resistance gene as no recombination could be detected between these markers and *Pi-l(t)* (Supplementary Fig S2)*.*

### Physical mapping and identification of resistance gene candidates

Based on chromosome landing of the flanking markers RM3246 and RRS19 on the genome of reference cultivar Nipponbare, the leaf blast resistance gene has been delimited to 6.19 Mb region spanning from position 9,095,272 to 15,287,816 bp close to centromere of chromosome 12 (Fig. 2). The search for gene content in the equivalent genomic region of reference cv. Nipponbare (http://rice.plantbiology.msu.edu) revealed the presence of 334 putative genes, of which five namely, *LOC_Os12g17090, LOC_Os12g17490, LOC_Os12g18360, LOC_Os12g18374* and *LOC_Os12g25170* had features of typical disease resistance genes encoding Nucleotide-binding site and leucine-rich repeat (NB-LRR) proteins (Table 2, Fig. 2). Two of these genes namely, *LOC_Os12g17090, and LOC_Os12g17490* represent the homologs of stripe rust resistance gene *Yr10*, while *LOC_Os12g18360* corresponds to a well known blast resistance *Pi-ta* gene that has been previously been reported to be present in Tetep, the progenitor of donor line RIL-4 . The locus *LOC_Os12g18374* corresponds to an allele of blast resistance gene *Pi-67(t)* gene that has previously been identified from Tetep^29^. All the five candidate resistance genes identified in the *Pi-l(t)* region are functional in rice genome as the expression evidences in the form of FL-cDNA transcripts and proteins have been detected for all the genes in the Rice Annotation Project database (http://rapd.dna.affrc.go.jp) (Table 2). The genomic position of the leaf blast resistance gene perfectly coincided with the genomic position of the neck blast resistance gene *Pb2* previously identified from the RIL4. Both the genes have been mapped to a 6.19 Mb region flanked by RM3246 and RRS19 and perfectly co-segregate with SSR markers RRS12, RRS16 and RRS17. These results strongly suggest that the same gene confers resistance to leaf and neck blast in RIL4.

**Table 2:** List of candidate resistance genes predicted at the region of *Pi-l(t)* locus in the genome sequence of cv. Nipponbare.

Sr No	Locus	Gene Coordinates (5'-3')	Gene size (bp)	Function	Expression support
1	*LOC_Os12g17090*	9,782,561..9,786,758	4802	Similar to stripe rust resistance protein Yr10 belonging to the nucleotide-binding site leucine-rich repeat (NBS-LRR) class	FL-cDNA (AK070311), Protein (Ac No. B9FJ98)
2	*LOC_Os12g17490*	10,016,692..10,021,620	4929	Similar to stripe rust resistance protein Yr10 belonging to the nucleotide-binding site leucine-rich repeat (NBS-LRR) class	FL-cDNA (AK065346), Protein (Ac No. Q2QU48)
3	*LOC_Os12g18360*	10,606,359..10,611,917	5559	Pi-ta protein belonging to the nucleotide-binding site leucine-rich repeat (NBS-LRR) class	FL-cDNA (AK071926), Protein (Ac No. Q9AY26)
4	*LOC_Os12g18374*	10,624,037..10,635,373	11,337	Similar to putative blast resistance gene *Pi-42(t)* belonging to the nucleotide-binding site leucine-rich repeat (NBS-LRR) class	FL-cDNA (AK072326), Protein (Ac No. B8BP46)
5	*LOC_Os12g25170*	14,449,900..14,451,648	1749	Similar to putative resistance gene belonging to nucleotide-binding site leucine-rich repeat (NBS-LRR) class	FL-cDNA (AK067669), Protein (Ac No. A2ZK34)

FL-cDNA: Full-length cDNA. The expression support for the candidate genes was ascertained by searching the TIGR database (http://rice.plantbiology.msu.edu) and the Rice Annotation Project database (RAP-db) (http://rapdb.dna.affrc.go.jp).

### Allelic relationship of resistance gene with ***Pita***^***2***^ and ***Pi67***

Previously two leaf blast resistance genes *Pita/Pita*^*2*^ (presumed to be as allelic or tightly linked) and *Pi67* have identified from the same genomic region of chromosome 12 of Tetep^29,35^. The Locus, *LOC_Os12g18374* which is one of the five candidates for leaf blast resistance gene identified from RIL4, has also been shortlisted as a candidate for leaf blast resistance gene *Pi-67* identified from TDH251^29^. Therefore, to see whether the leaf blast resistance gene identified from RIL4 is identical to *Pita/Pita2* and *Pi-67* genes, its allelic relationship with these genes was studied by observing the segregation of leaf blast resistance in F_2_ progenies of crosses of RIL4 with IRBLta2-Pi and TDH251, respectively. No susceptible individual was found among the F_2_ progenies of crosses RIL4 x TDH251 and RIL4 x IRBLta2-Pi thereby suggesting that the leaf blast resistance gene in RIL4 is either identical or tightly linked to *Pita/Pita2* or *Pi67* (Table 3).

**Table 3:** Reaction to *Pyricularia oryzae* isolate Po-HPU2216 5–2 of F_2_ progenies of crosses of RIL4 with TDH251 and IRBLta2-Pi.

Genotype/cross	No. of plants (R:S) ^b^	Expected ratio (R: S)	χ^2^	*P* value
Parental genotypes^*a*^				
RIL4 (*Pi-l(t*))	10: 0	1 : 0	–	–
TDH251(*Pi67*)	10: 0	1 : 0	–	–
IRBLta2-Pi (*Pita*^*2*^)	10: 0	1 : 0	–	–
HPU2216 (*Pita*)	0 :10	0 :1	–	–
Cross				
F_2_ (RIL4 x TDH251)	752: 0	15 : 1	50.13	< 0.001
F_2_ (RIL4 x IRBLta2-Pi)	950: 0	15 : 1	63.32	< 0.001

^a^The known resistance genes in parental genotypes are given in parenthesis.

^b^R = Resistant (reaction type 0–2); S = susceptible (reaction type 3–4). χ^2^ = the actual value of Chi-square test for susceptible / resistant ratio.

## Discussion

The blast disease affects rice crop from seedling to reproductive stage thus necessitating the identification of all-stage effective resistance genes that could provide protection throughout the crop season. A great majority of blast resistance genes identified till date have been identified through phenotypic analysis of progenies of different crosses for leaf blast resistance. Owing to inherent difficulties associated with phenotyping of neck/panicle blast resistance, a few major genes mediating protection to neck blast have been identified; of some 118 major blast resistance genes identified from rice, only three, *Pb1*, *Pi25* and *Pi64,* are known to provide resistance to panicle and neck blast^15–17^. There has been a difference of opinion among the rice researchers regarding the relationship between neck and leaf blast resistance genes. While some researchers have argued that the mechanisms of leaf and neck blast resistance are different based on the fact that genotypes showing resistance to leaf blast in some instances are susceptible to neck blast and *vice versa*^18,19,36^. Others have inferred that same genes are involved in providing resistance against both phases of the disease^17,37^.

Similar to our results, two more blast resistance genes *Pi25(t)* and *Pi64* have previously been shown to confer resistance to both leaf and neck blast, and in many instances the gene/QTLs for panicle blast resistance have co-localized with major leaf blast resistance genes^37^, thereby suggesting the involvement of common genes for resistance to both leaf and neck blast.

The leaf blast resistance gene identified in RIL4 has been mapped to a recombination suppressed region near the centromere of chromosome 12 from where three blast resistance genes, namely, *Pita*, *Pita2* and *Pi67* have previously been identified in Tetep, the resistance donor for line RIL4^29,35,38^. The fact that the rice variety HPU2216, used as a susceptible parent in our study, contains *Pita* gene inherited from a popular rice variety IR36 suggests that this gene is not involved in blast resistance under our conditions. On the other hand, the lack of segregation of resistance in F_2_ progenies of crosses of RIL4 with IRBLta2-Pi and TDH251 has suggested that the leaf blast resistance gene in RIL4 is either allelic or tightly linked to *Pita2* and *Pi67* genes previously identified from the same region^29,35^.

The genomic region close to centromere of chromosome 12 is known to harbor a cluster of blast resistance genes and nearly 19 allelic and/or closely linked blast resistance genes have been mapped to this region in different rice genotypes^39,40^. The same genomic region in Tetep and other broad-spectrum resistance genotype Tadukan has been shown to harbor three blast resistance genes *Pita*, *Pita*^*2*^, *Pi67*^29,35,38^. The gene *Pita* was the first blast resistance genes to be identified from the region followed by *Pita*^*2*^^24,25^. Subsequent genetic studies have indicated that *Pita* and *Pita*^*2*^ are allelic or closely linked^38^. Literature reports have consistently suggested that the *Pita*^*2*^ exhibits a broader spectrum of resistance compared to *Pita*, the former controlling all the strains controlled by *Pita* plus additional strains that are virulent to *Pita*; till date no isolate that is virulent on *Pita*^*2*^ and avirulent to *Pita* has been recorded^25,35,38^. These observations have been taken to reflect that the *Pita*^*2*^ specificity is a combination of at least two R genes- *Pita* plus a second linked R-gene^38,41^. All the *Pita*^*2*^ rice varieties analyzed till date like Tetep, Reiho, Katy and isogenic lines namely, IRBLta2-Re and IRBLta2-Pi are reported to contain resistant *Pita* allele suggesting that *Pita* is required for resistance function of *Pita*^*2*^^38,41^. Another gene *Ptr* located in the linkage block near the centromere of Chromosome 12 has been identified in the U.S. tropical *japonica* cultivar Katy harboring *Pita*/*Pita*^*2*^genes inherited from the Tetep. The mutants with defects in *Ptr* gene are compromised in *Pita* and *Pita*^*2*^ resistance thereby indicating that the gene is involved in transduction of defense signaling in *Pita/Pita*^*2*^-mediated resistance pathway^32^. The *Ptr* gene has been cloned recently and shown to encode a protein with an Armadillo (ARM) repeat domain^41^. Interestingly, all the rice varieties identified to display *Pita*^*2*^ resistance till date invariably harbor a Katy type resistant *Ptr* allele in addition to having resistant *Pita* allele^41^. The rice variety Yashiro-mochi in which *Pita* gene was initially cloned^38^ and few other *Pita* rice varieties like Pi No.1, Pi No.2 and YT14 incidentally do not harbor resistant *Ptr* haplotype similar to *Pita*^*2*^ rice varieties^41^. These data suggest that *Pita*^*2*^ resistance specificity is conditioned by a combination of *Pita*, *Ptr* and yet unknown R-gene that probably recognizes the isolates virulent on *Pita* gene, while the *Pita* mediated resistance in varieties like Yashiro-mochi operates through a different pathway that does not require *Ptr* protein as a component of defense signaling pathway.

The largest class of *R* genes in plants belongs to the conserved family of nucleotide-binding site leucine–rich repeat (*NBS-LRR*) genes that play role in detecting pathogen effectors (avirulence proteins) and activate defense signaling. Approximately 500 *NBS-LRR* genes have been predicted in the rice genome, and all the major blast resistance genes cloned until date, except for *Pid2*, belong to this class^39^. Although the majority of these genes were identified as single loci that follow Flor’s gene-for-gene model, the blast resistance in several cases has been shown to be mediated by functional pairs of *NBS-LRR* genes as seen for resistances mediated by *Pia*, *Pi5* and *Pik*^42–44^. Such *NBS-LRR* gene pairs show extremely tight physical linkage and are arranged in inverted orientation suggesting a common mechanism of their action^45^. In such gene pairs, one member known as “sensor” functions to negatively regulate the other member known as “helper” in an inactive complex to prevent autoimmunity in the absence of pathogen, and binding of the pathogen *AVR* gene encoded effectors triggers the release of this negative regulation allowing the helper to activate downstream defense signaling^46^. Autoactive *NBS-LRR* helper and their negative regulators are expected to function as a single unit and likely to remain genetically linked^47^. Further, emerging evidences in different crops suggest that *NBS-LRR* genes form genetic networks in which a single helper gene can interact with different sensors that confer immunity to diverse pathogens e.g. a single helper NRC4 is required for the function of several sensors *NBS-LRR* genes that confer immunity to oomyctes, bacteria, viruses nematodes and insects^47^.

Genome wide analysis of *NBS-LRR* genes in Tetep has also revealed the presence of 43 *NBS-LRR* gene pairs comprising of closely linked members that are arranged in head-to-head orientation in the genome^21^. One such pair of *NBS-LRR* genes represented by functional genes *LOC_Os12g18360* and *LOC_Os12g18374* is also located in the Tetep derived region associated with leaf and neck blast resistance in RIL4. The gene locus *LOC_Os12g18360* corresponds to the blast resistance gene *Pita,* while *LOC_Os12g18374* is reported to be a candidate for two different leaf blast resistance genes, *Pi67* in Tetep^29^ and *Pi42* in rice genotype DHR9^33^. The CRISPR/Cas9 knockouts of *LOC_Os12g18360* (*Pita*) in transgenic lines expressing matching partner *LOC_Os12g18374* are also reported to exhibit auto-immunity thus suggesting that the two genes work as a sensor-helper pair^21^.

Taking cue from the findings of earlier studies on *Pita*^*2*^ gene^25,35,38,41^ and current developments in understanding of disease resistance in plants^21,46,47^, we propose that *Pita*^*2*^ resistance specificity is conditioned by at least three tightly linked *NBS-LRR* genes. Among these, the helper encoded by *LOC_Os12g18374* interacts with two different sensors, one encoded by gene *LOC_Os12g18360* (*Pita*) which retains recognition specificity for effectors of *Pita* avirulent isolates, and other encoded by one of three closely linked *NBS-LRR* genes located in the *Pi-l(t)* region which recognizes the effectors of *Pita* virulent isolates*.* Further studies involving generation of gene knockouts of candidate *NBS-LRR* genes in RIL4 and testing their resistance spectrum against a diverse collection of *Pita* and *Pita*^*2*^ specific blast isolates are required to explain the molecular basis of *Pita*^*2*^ resistance specificity. The mapping of leaf blast resistance gene in RIL4 to the same Tetep derived genomic region as was previously shown to be associated with neck blast resistance suggested that the same gene (*Pi-l(t)* = *Pb2* = *Pita*^*2*^), confers resistance to both phases of disease. The gene is located in a recombination suppressed region harboring a cluster of *NBS-LRR* genes that all are functionally active in rice genome as evinced by FL-cDNA and protein expression support for these genes in Nipponbare genome. The gene along with its linked *NBS-LRR* genes can be effectively incorporated into susceptible genetic backgrounds using co-segregating markers RRS12, RRS77 and RRS16 to develop leaf and neck blast resistant varieties.

## Methods

### Plant material and resistance phenotyping

An *indica* rice variety, HPU2216, containing blast resistance gene *Pita* was used as a susceptible parent. The variety is derived from the cross IR8/IR 2053–521-1–1//IR36 and inherit its *Pita* gene from the popular IRRI bred rice variety IR36. RIL4, an F_2:12_ recombinant inbred derivative of cross HPU2216 x Tetep from which a neck blast resistance gene *Pb2* has been mapped previously^30^ was used as a resistant parent. An F_2_ population derived from the cross of HPU2216 with RIL4 was used for genetic studies and mapping of leaf blast resistance gene. The seeds of parental genotypes and F_2_ population were sown in a plastic trays (40 × 25 × 10 cm) filled with potting mixture (soil: sand: 3: 1) and grown in a growth chamber maintained at 25 to 30 °C for 21 days.

The *P. oryzae* isolate, *Po-HPU2216-5–2* (race U63-i0-k167-z04-ta023), which is virulent on HPU2216 and avirulent on RIL4 and Tetep was used for the phenotypic screening of leaf blast resistance. The virulence spectrum of the isolate on monogenic blast differentials is provided in Supplementary Table S2. Altogether 934 F_2_ seedlings of cross HPU2216 x RIL4 were spray inoculated with the culture of this isolate. The procedure described by Rathour et al*.*^48^ was used for the preparation of inoculum and disease scoring. Briefly, mycelia from 10-day old cultures were macerated in 5 ml of distilled water and plated onto Mathur’s medium^49^ for sporulation in Petri plates. After 8 to 10 days of incubation at 25 ± 1 °C, the plates were washed with 10 ml of distilled water to make spore suspension, which was filtered through two layers of muslin cloth and spore concentration adjusted to 40–50 conidia/microscopic field (40 X). About 30–40 ml of the spore suspension was sprayed on 21-day-old seedlings using a glass atomizer. The inoculated seedlings were kept in a humidity chamber at 25 ± 1 °C and sprayed three to four times a day with distilled water to maintain high humidity. Disease reaction was recorded after 7 days of inoculation using 0–5 scale given by Mackill and Bonman^26^. The number of resistant and susceptible F_2_ seedlings was recorded and the data subjected to Chi-square analysis to test the goodness of fit to Mendelian ratios. The individual seedlings showing highly resistant (0–1 score) or susceptible (4–5 score) reactions were transferred to pots for later use in extraction of DNA. The DNA from 220 susceptible F_2_ plants was used for the mapping of the resistance gene using recessive class analysis^50^.

### Mapping of leaf blast resistance gene

Genomic DNA of the parents and F_2_ seedlings was isolated using the standard CTAB method^51^. Initially, two polymorphic markers STS-5 and RM1261, one each from the short and long arm of chromosome 12, were tested for their linkage to leaf blast resistance locus using recessive class approach (RCA)^50^. Since the initial linkage analysis indicated STS-5 and RM1261 to be the flanking markers for the resistance locus, six additional polymorphic SSR markers viz., RM3246, RRRS12, RRS77, RRS16, RRS19 and RRS69 were selected from the genomic region bracketed by these markers for chromosome walking to the resistance gene. These internal markers were used for the genotyping of the recombinants detected with STS-5 and RM1261 to delimit the genomic region for the resistance locus. The primer sequences for the markers used in mapping were adapted from the web site of the International Rice Microsatellite Initiative (IRMI; http://www.gramene.org) and Kumar et al*.*^33^.

DNA amplification was carried out in a 12.5 µl reaction volume containing 20 ng template DNA, 0.2 mm of each dNTP, 0.2 µM of each primer, 1.5 mm MgCl_2,_ 1X PCR buffer (10 mm Tris–HCl, 50 mm KCl, pH 8.3) and 1 unit *Taq* polymerase (Gotaq® DNA Polymerase, Promega). PCR amplification was carried out in a thermocycler (Proflex PCR System; Applied Biosystems, Life Technologies, USA) using the following temperature profile: initial denaturation at 94 °C for 5 min followed by 39 cycles at 94 °C for 30 s, 55 °C for 30 s, 72 °C for 45 s and a final extension at 72 °C for 5 min followed by rapid cooling to 4 °C. The PCR products were resolved in 4% agarose gel and visualized by ethidium bromide staining. The intensity of linkage between the markers and resistance gene(s) was deduced by the segregation analysis of 220 susceptible F_2_ plants. Linkage analysis was performed with Map Disto Version 2.0 software^52^ and the recombination frequency was converted to map distance expressed in cm (centi Morgan) using the Kosambi function^53^.

### Delineation of the physical position of the blast resistance locus and identification of candidate resistance gene(s)

The physical map of the resistance locus was constructed in silico by aligning the sequences of flanking markers on the genome sequence of cv. Nipponbare using BLAST programme of Rice Annotation Project Database (*RAP-DB*) (http://rapdb.dna.affrc.go.jp/). The gene content in the target region was deduced by searching the TIGR Rice Genome Annotation Project database of cv. Nipponbare (http://rice.plantbiology.msu.edu). The gene(s) encoding nucleotide-binding site and leucine-rich repeat (NBS-LRR) proteins were shortlisted as likely candidates for the blast resistance gene identified from RIL4. The functional support for the identified candidate genes was ascertained by searching for the presence of matching full length c-DNA (FL-cDNA) transcripts and proteins in the TIGR and the Rice Annotation Project databases (http://rapdb.dna.affrc.go.jp/; http://rice.plantbiology.msu.edu).

### Allelic tests with ***Pita***^***2***^ and ***Pi67*** resistance genes

The allelic relationship of the leaf blast resistance gene identified from RIL4 with blast resistance genes *Pita*^*2*^ and *Pi67* was studied by crossing the line RIL4 with genotypes IRBLta2-Pi and TDH251 harboring these genes^29,54^. The seed of line IRBLta2-Pi was kindly provided by Dr. N. Kobayashi, International Rice Research Institute, Philippines, while for TDH251, the seed available in our department was used. The F_2_ progenies of crosses RIL4 x IRBLta2-Pi and RIL4 x TDH251 along with parental genotypes were inoculated with the blast isolate, *PO-HPU2216-5–2*. The procedure for disease inoculation and scoring was essentially similar to that described in the preceding sections. The observed ratio of Resistant : Susceptible plants in each cross was tested for goodness of fit to 15:1 ratio excepted for duplicate independently segregating dominant genes using Chi-square test.

### Ethical declarations

This study does not involve any ethical issues. There were no human or animal subjects involved in the study.

### Statement regarding plant materials

The monogenic lines including IRBLta2-Pi have been procured from IRRI, Philippines under material transfer agreement. Further, all plant experiments involved in this study have been conducted in accordance to relevant regulations and guidelines.

### Supplementary Information

Supplementary Information.

## Data Availability

All the data used in the present study are included in this manuscript and supplementary information files. The genomic resources of *Oryza sativa* ssp. *japonica* cv. Nipponbare used for the construction of physical map, searching gene content and full length Transcripts (Fl-cDNA) and proteins sequences are available in public domain at http://rapdb.dna.affrc.go.jp and http://rice.plantbiology.msu.edu.
