# Supplementary material for: Genetic and molecular analysis of leaf blast resistance in Tetep derived line RIL4 and its relationship to genes at Pita/Pita2 locus
Source: Sci Rep. 2023 Oct 31;13:18683. doi: 10.1038/s41598-023-46070-7 (PMC10618204; doi:10.1038/s41598-023-46070-7)

Supplementary material

Genetic and molecular analysis of leaf blast resistance in Tetep derived line RIL4 and its relationship to genes at *Pita/Pita*^{2} locus

B. Biswas, K. Thakur, T. D. Pote, K. D. Sharma, S. Gopala Krishnan, A. K. Singh, T. R. Sharma & R. Rathour

Supplementary Table S1. Number of recombinants detected between the leaf blast resistance gene and different markers of chromosome 12 among 220 leaf blast susceptible plants of cross HPU2216 X RIL4.

Marker	No. of recombinants		Total recombination events
	Single cross over	Double cross over	
**STS-5**	9	2	**13**
**RM 3246**	9	-	**9**
**RRS 12**	0	-	**0**
**RRS 77**	0	-	**0**
**RRS 16**	0	-	**0**
**RRS 19**	2	-	**2**
**RRS 69**	3	-	**3**
**RM1261**	7	-	**7**

Supplementary Table S2. Virulence reaction of *Pyricularia oryzae* isolate Po-HPU2216-5-2 on parental genotypes and rice monogenic lines harbouring different blast resistance genes.

Sr. No.	Genotype	Known gene	Chromosome	Reaction
**Monogenic lines**				
1	IRBLsh-S	*Pi-sh*	1	R
2	IRBLb-B	*Pi-b*	2	S
3	IRBLt-K59	*Pi-t*	1	S
4	LTH	*?*	-	S
5	IRBLa-A	*Pi-a*	11	S
6	IRBLi-F5	*Pi-i*	9	R
7	IRBL3-CP4	*Pi-3*	9	R
8	IRBL5-M	*Pi-5 (t)*	9	R
9	IRBLks-F5	$Pi\text{-}k^{s}$	11	S
10	IRBLkm-Ts	$Pi\text{-}k^{m}$	11	R
11	IRBL1-CL	*Pi-l*	11	S
12	IRBLkh-K3	$Pi\text{-}k^{h}$	11	S
13	IRBLk-KA	*Pi-k*	11	S
14	IRBLkp-K60	$Pi\text{-}k^{p}$	11	S
15	IRBL7-M	*Pi-7 (t)*	11	S
16	IRBL9-W	*Pi-9*	6	R
17	IRBLz-Fu	*Pi-z*	6	R
18	IRBLz^{5}-CA-1	$Pi\text{-}z^{5}$ *(Pi-2)*	6	R
19	IRBLz^{t}-T	$Pi\text{-}z^{t}$	6	S
20	IRBLta^{2}-Pi	$Pi\text{-}ta^{2}$	12	R
21	IRBLta^{2}-Re	$Pi\text{-}ta^{2}$	12	R
22	IRBL12-M	*Pi-12 (t)*	12	R
23	IRBLta-K1	*Pi-ta*	12	R
24	IRBLta-CP1	*Pi-ta*	12	S
25	IRBL19-A	*Pi-19*	12	S
26	IRBL20-IR24	*Pi-20*	12	S
27	IRBL11-Zh	*Pi-11 (t)*		S
28	TDH251	*Pi67(t)*	12	R
**Parental genotypes**				
1	HPU2216	*Pi-ta*	-	S
2	RIL4	*Pb2*	-	R
3	Tetep	*Pi-1, Pi54,* *Pita,* $Pi\text{-}ta^{2}$	11, 11, 12, 12	R

R=Resistant reaction (reaction type 0-2); S= Susceptible (reaction type 3-5).

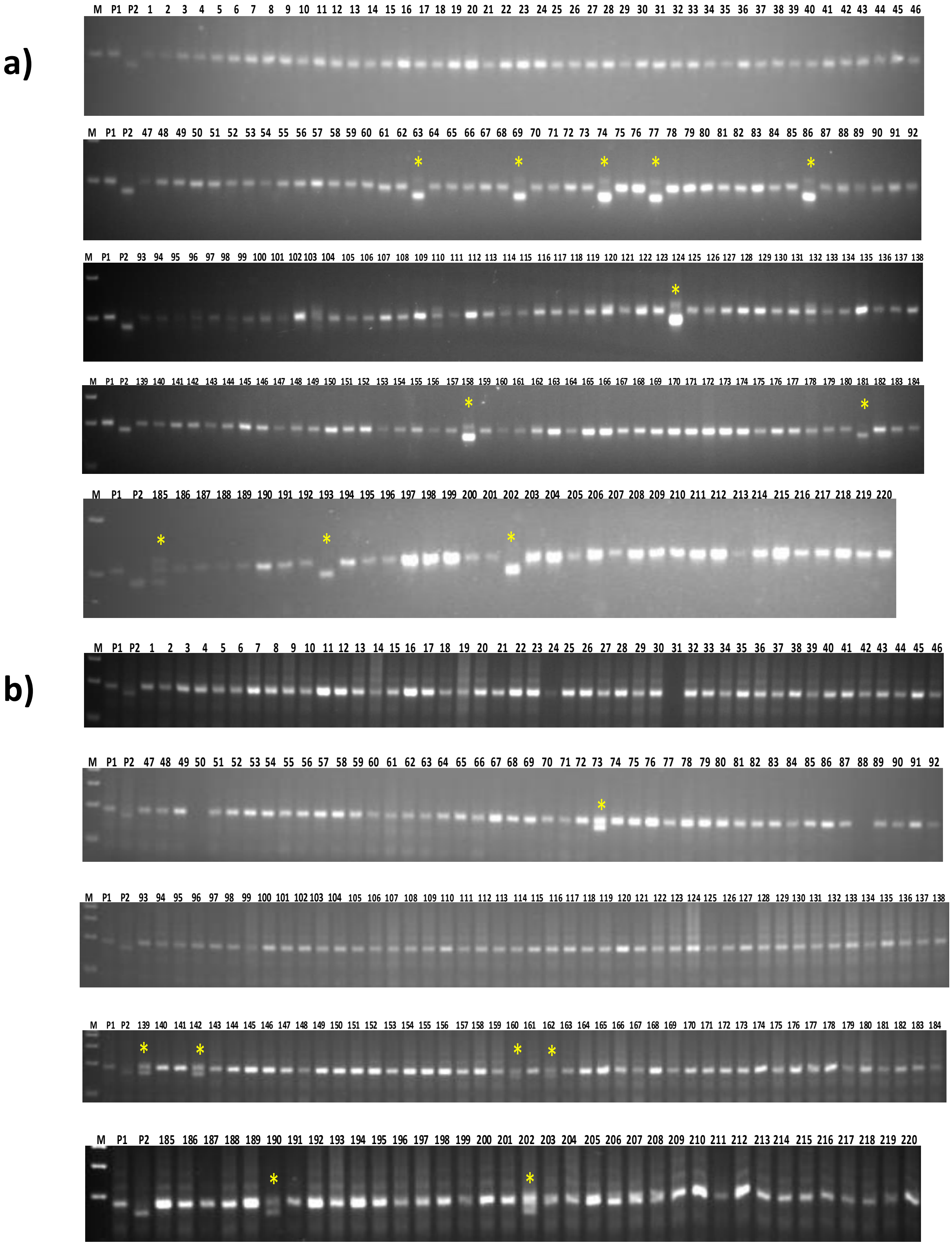

Supplementary Figure S1. Genotyping of 220 leaf blast susceptible F_2 plants of cross HPU2216 x RIL4 with marker a) STS-5 and b) RM1261 . P1= HPU2216; P2= RIL4. *Plants marked by asterisk represent the recombinants.*

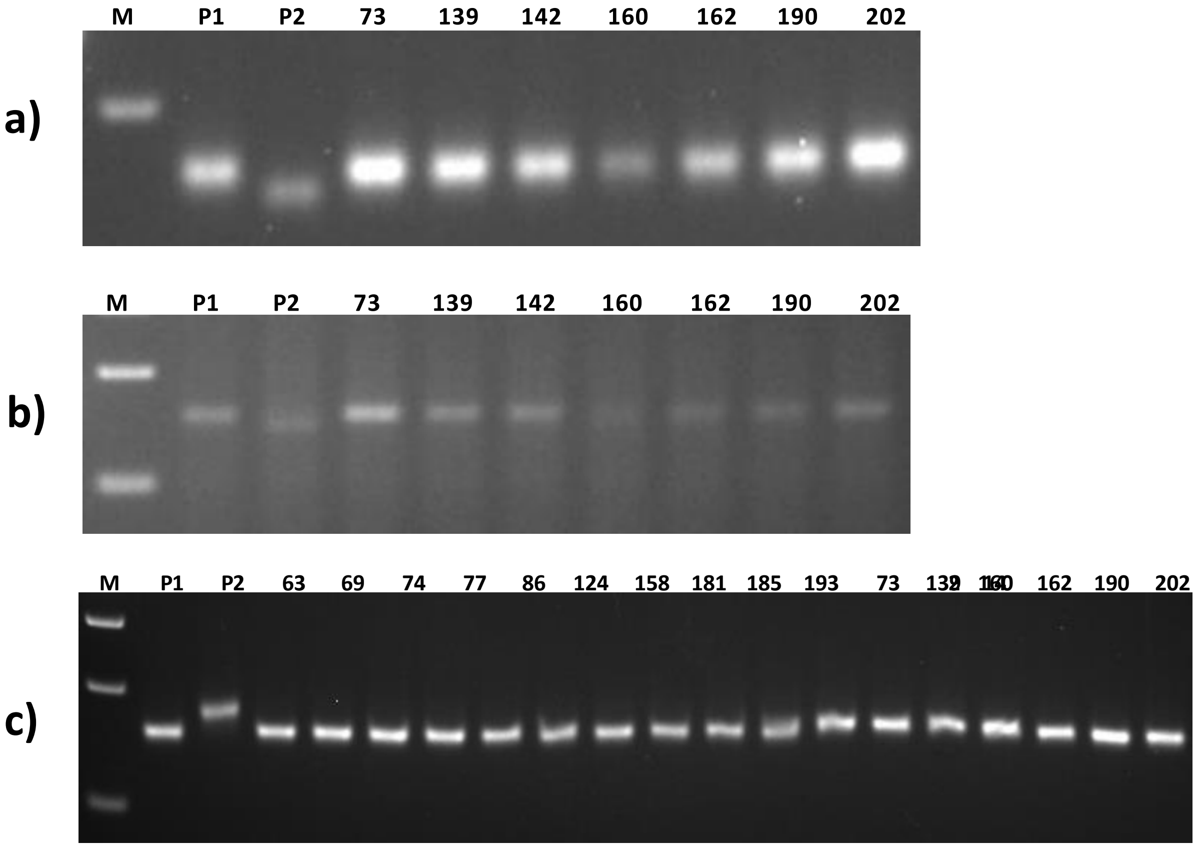

Supplementary Figure S2. Genotyping of the recombinants detected with STS-5 and RM1261 with internal polymorphic markers. A) Genotyping of recombinants detected with RM1261 with RRS77. B) Genotyping of recombinants detected with RM1261 with RRS16. C) Genotyping of recombinants detected with STS-5 and RM1261 with RRS12. P1= HPU2216; P2= RIL4.

Original gels

Supplementary Figure S1. Genotyping of 220 leaf blast susceptible F_2 plants of cross HPU2216 x RIL4 with marker a) STS-5 and b) RM1261 . P1= HPU2216; P2= RIL4. *Plants marked by asterisk represent the recombinants.*

a) **Genotyping of 220 leaf blast susceptible F_2 plants of cross HPU2216 x RIL4 with marker STS-5**

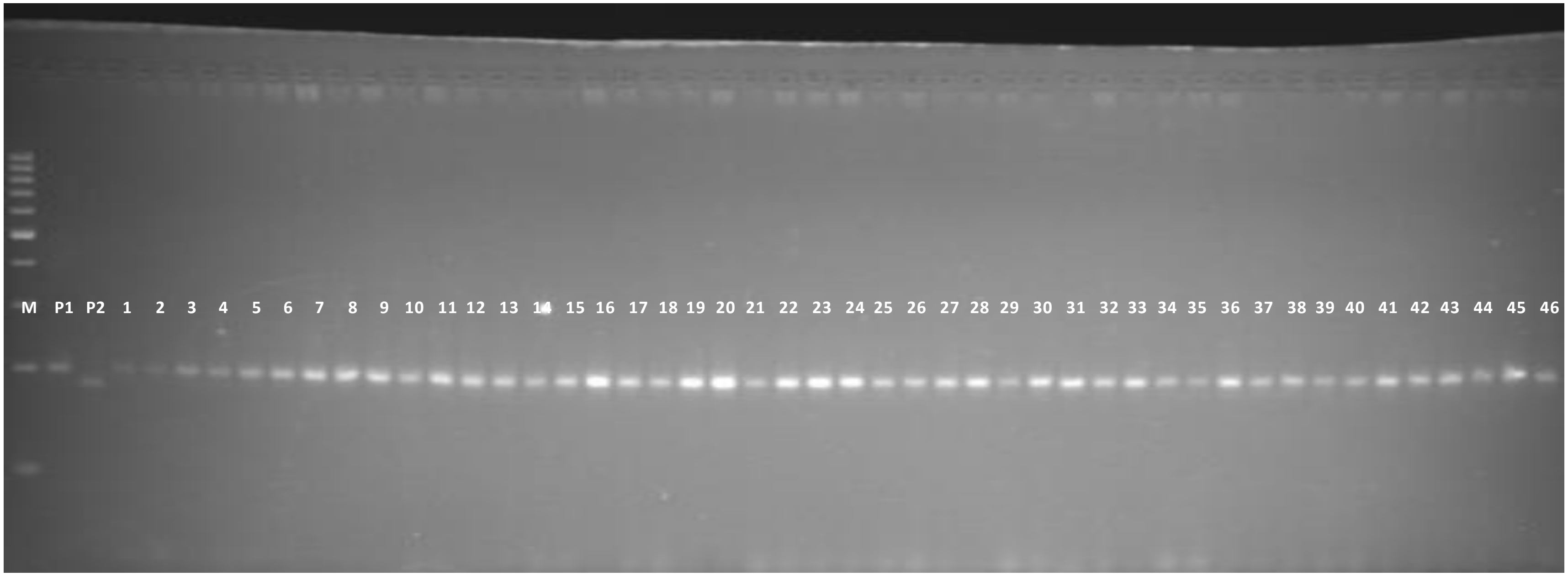

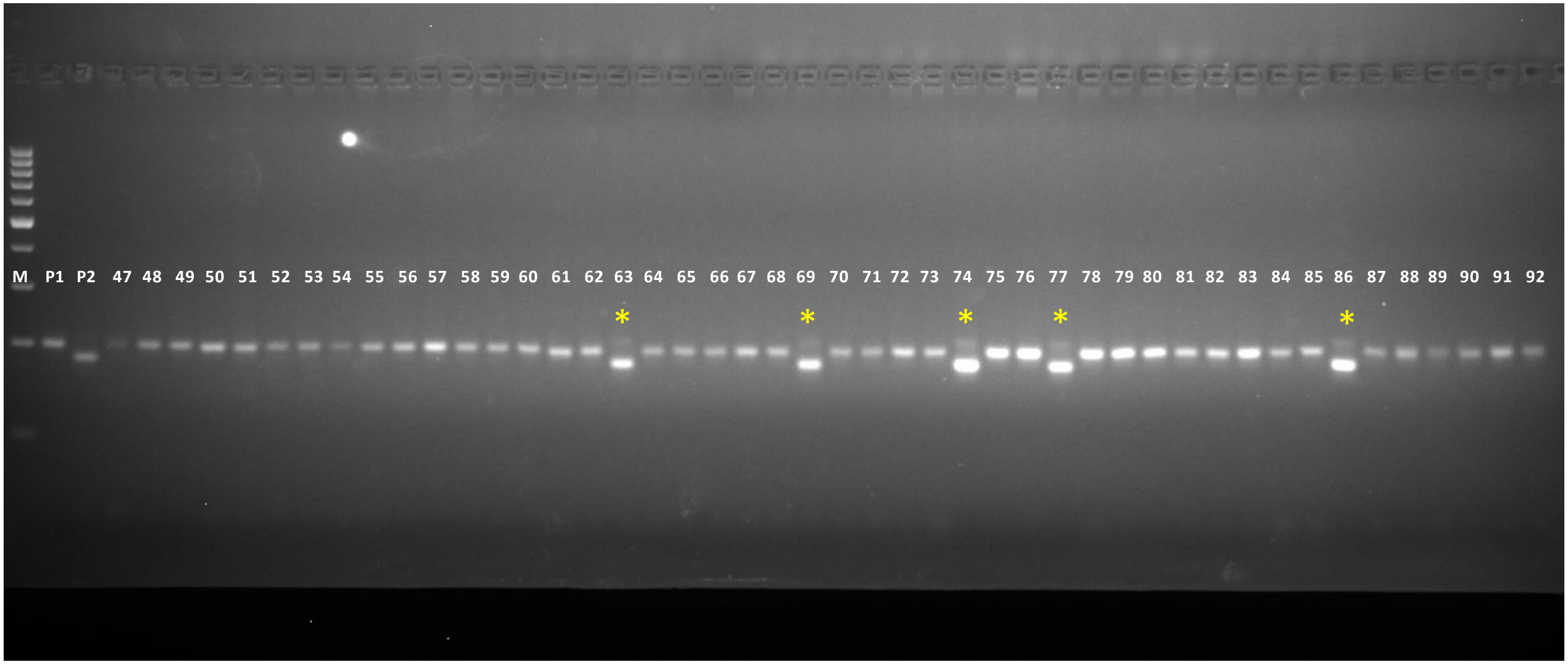
M P1 P2 47 48 49 50 51 52 53 54 55 56 57 58 59 60 61 62 63 64 65 66 67 68 69 70 71 72 73 74 75 76 77 78 79 80 81 82 83 84 85 86 87 88 89 90 91 92
* * * * *

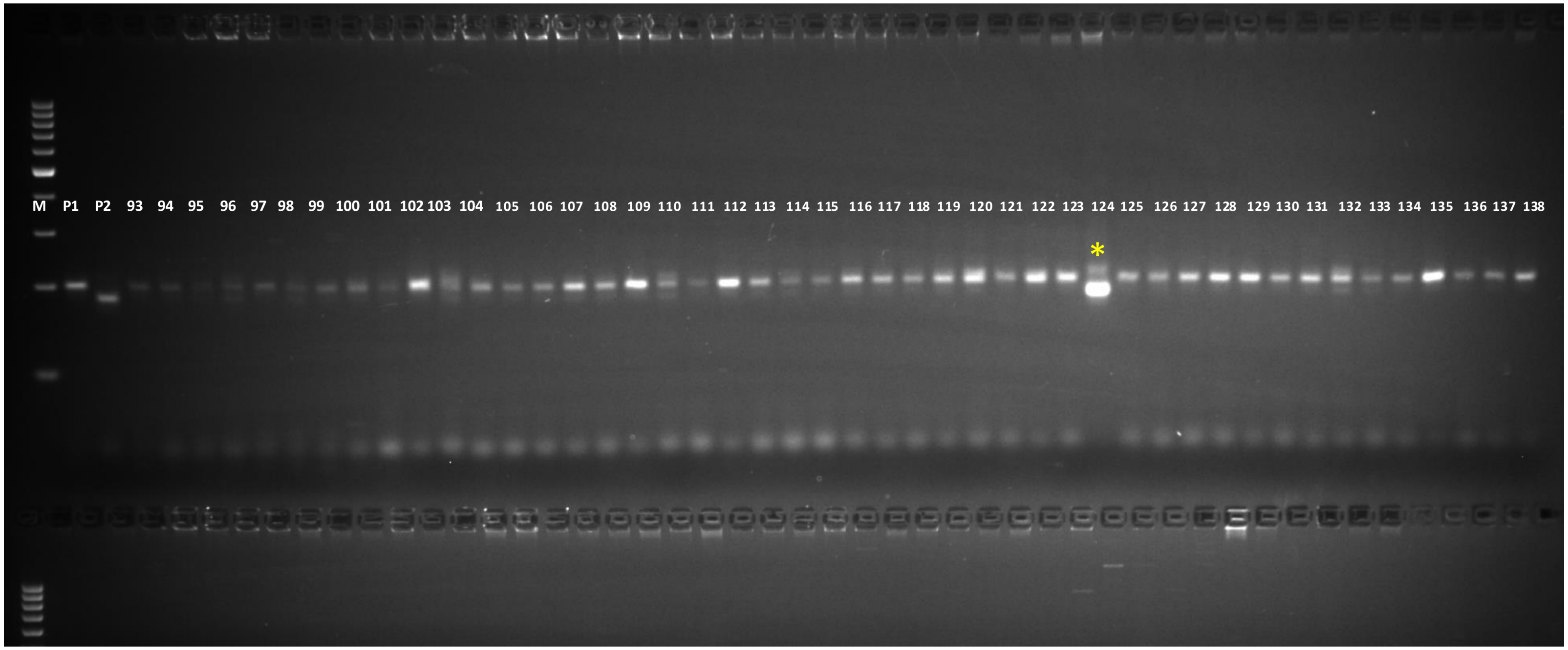
M P1 P2 93 94 95 96 97 98 99 100 101 102 103 104 105 106 107 108 109 110 111 112 113 114 115 116 117 118 119 120 121 122 123 124 125 126 127 128 129 130 131 132 133 134 135 136 137 138
*

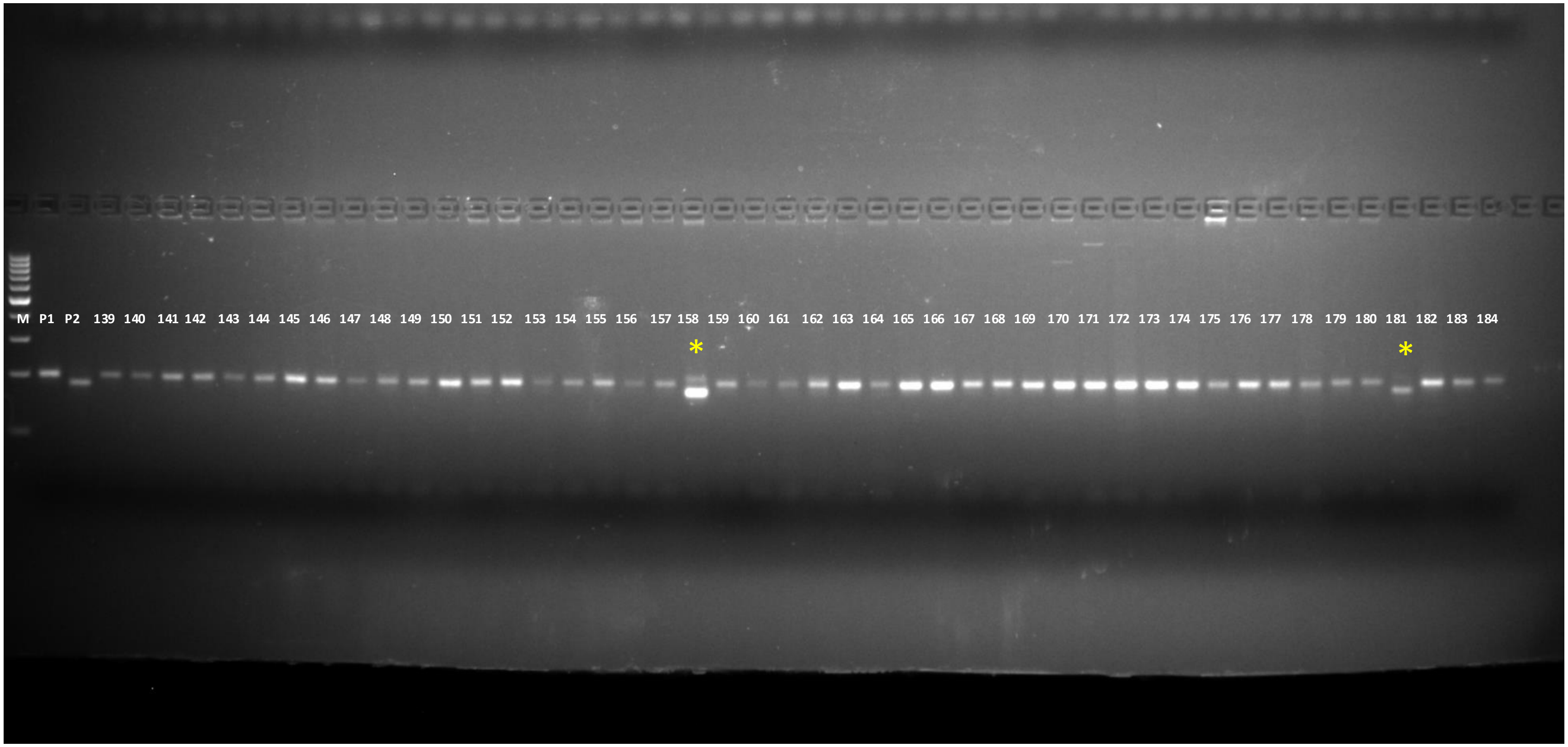
M P1 P2 139 140 141 142 143 144 145 146 147 148 149 150 151 152 153 154 155 156 157 158 159 160 161 162 163 164 165 166 167 168 169 170 171 172 173 174 175 176 177 178 179 180 181 182 183 184
*
*

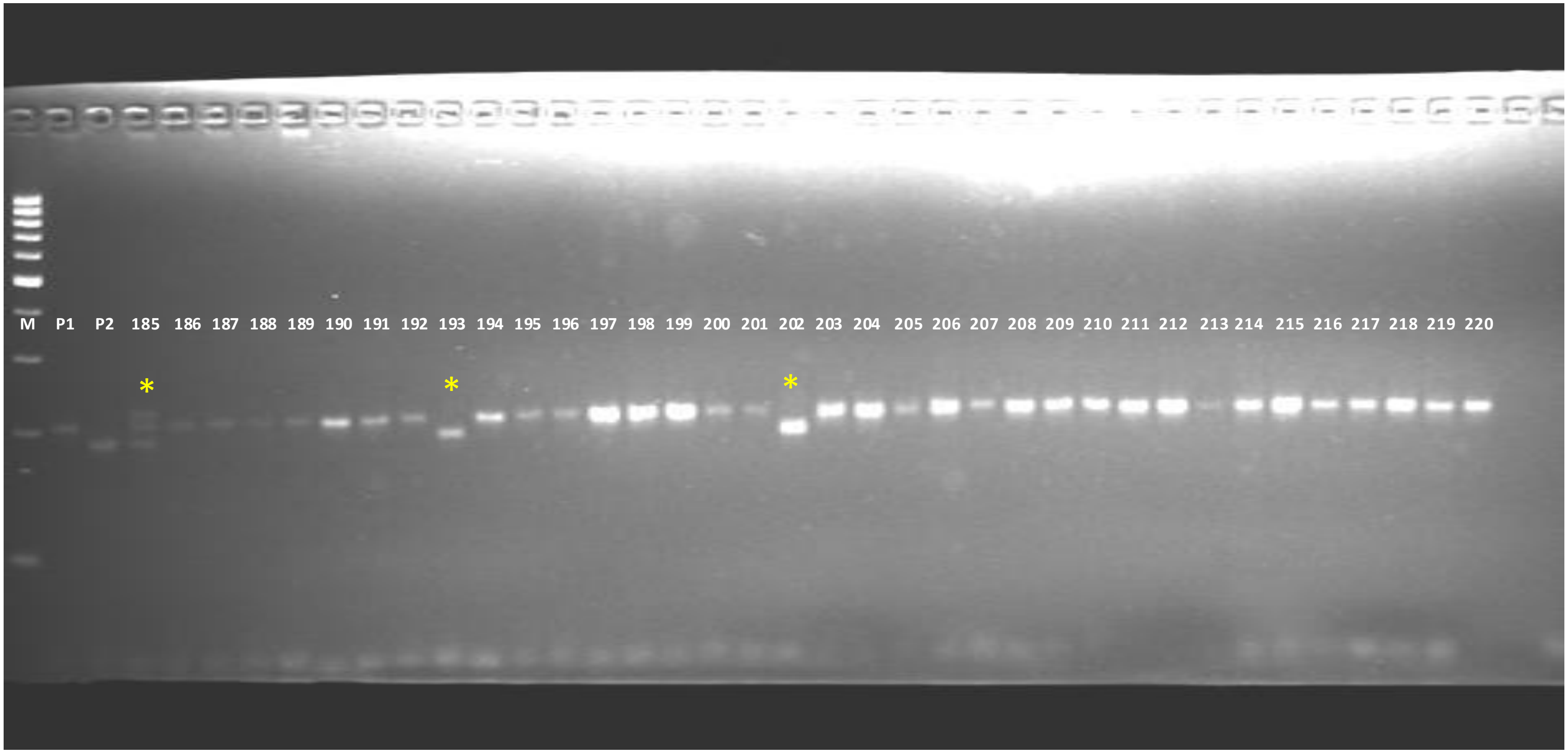
M P1 P2 185 186 187 188 189 190 191 192 193 194 195 196 197 198 199 200 201 202 203 204 205 206 207 208 209 210 211 212 213 214 215 216 217 218 219 220
*
*
*

b) Genotyping of 220 leaf blast susceptible F_2 plants of cross HPU2216 x RIL4 with marker RM1261

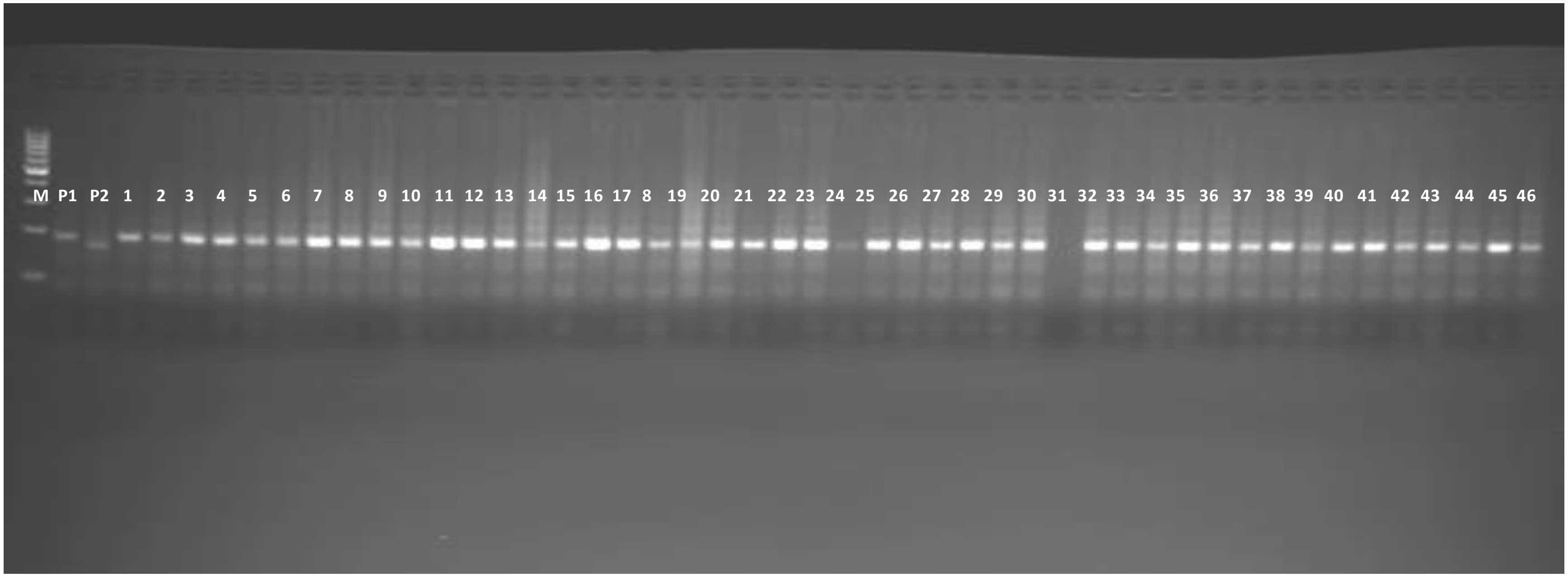

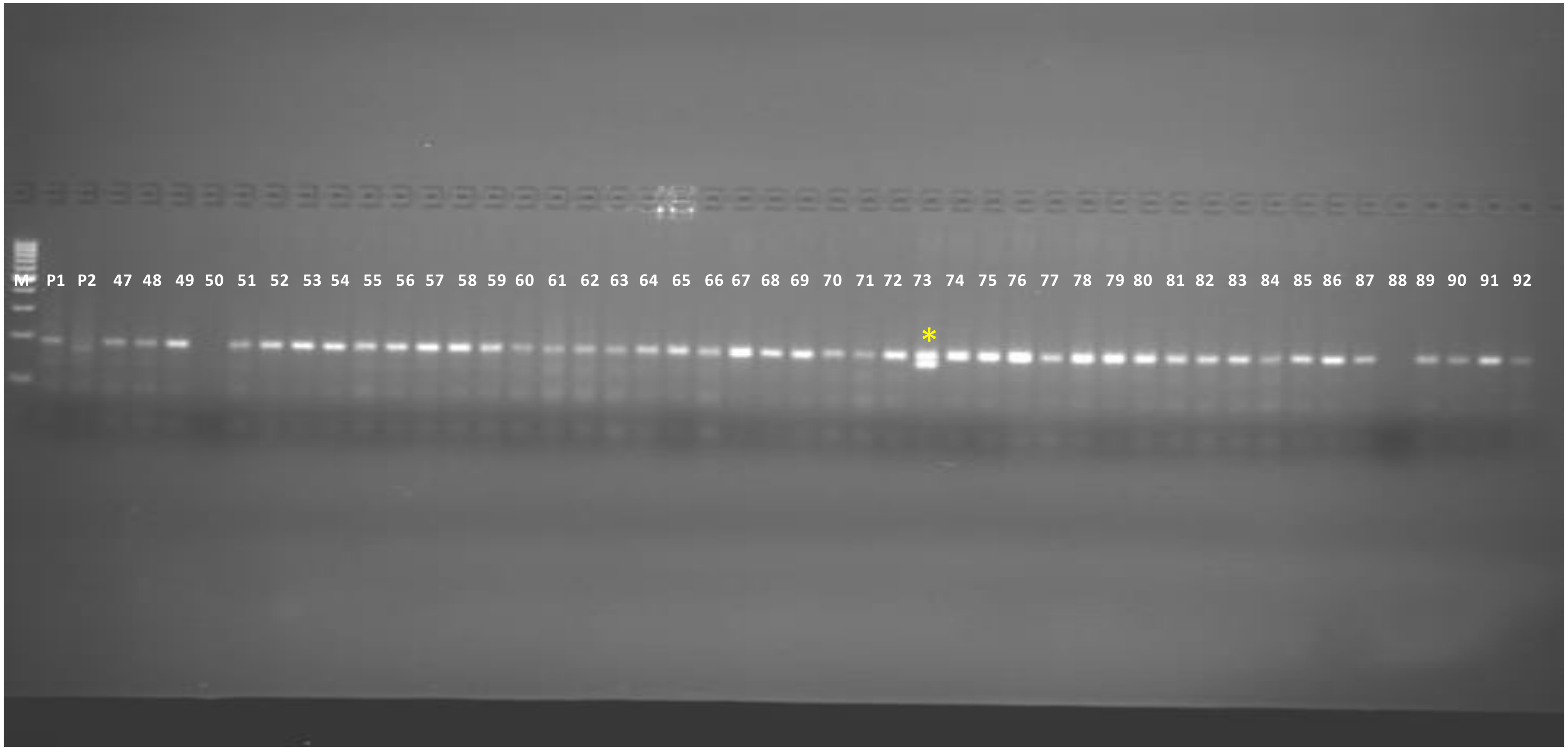
M P1 P2 47 48 49 50 51 52 53 54 55 56 57 58 59 60 61 62 63 64 65 66 67 68 69 70 71 72 73 74 75 76 77 78 79 80 81 82 83 84 85 86 87 88 89 90 91 92
*

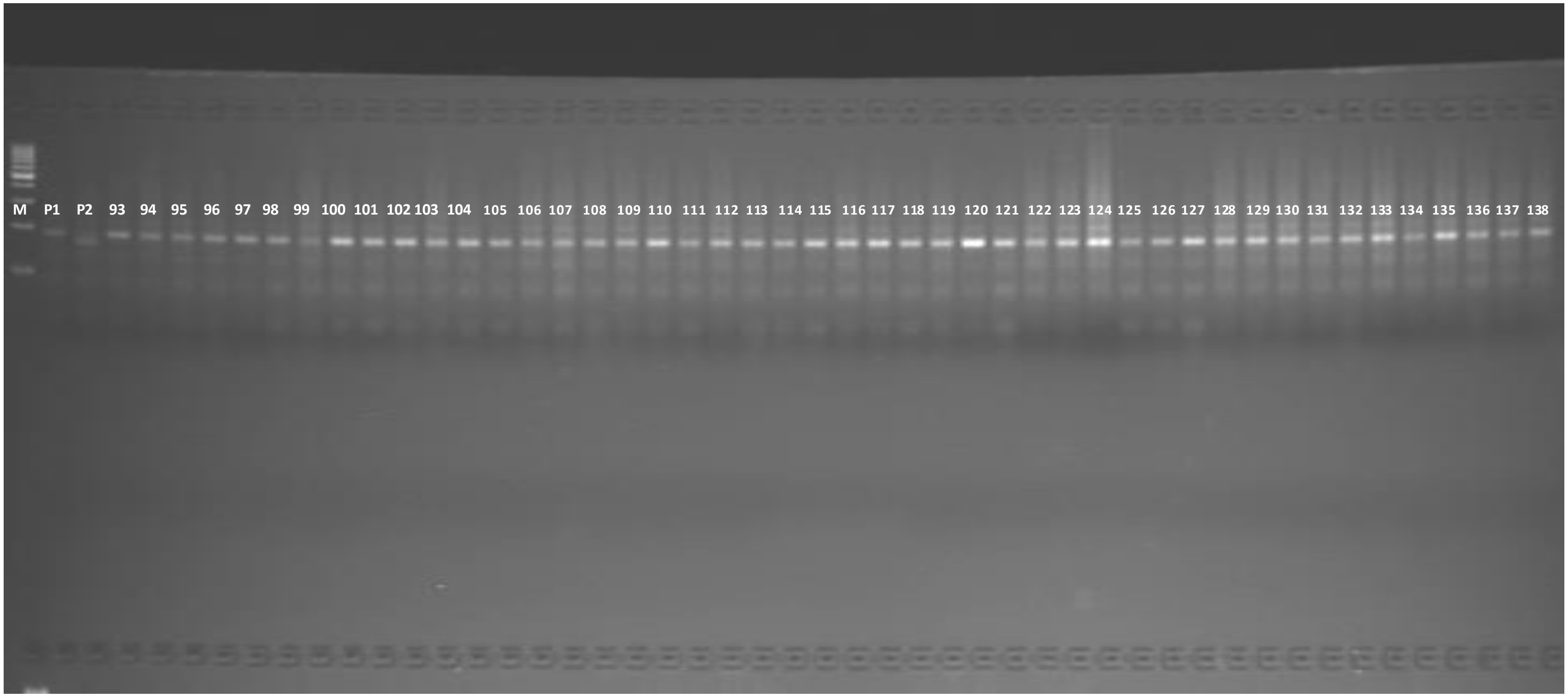
M P1 P2 93 94 95 96 97 98 99 100 101 102 103 104 105 106 107 108 109 110 111 112 113 114 115 116 117 118 119 120 121 122 123 124 125 126 127 128 129 130 131 132 133 134 135 136 137 138

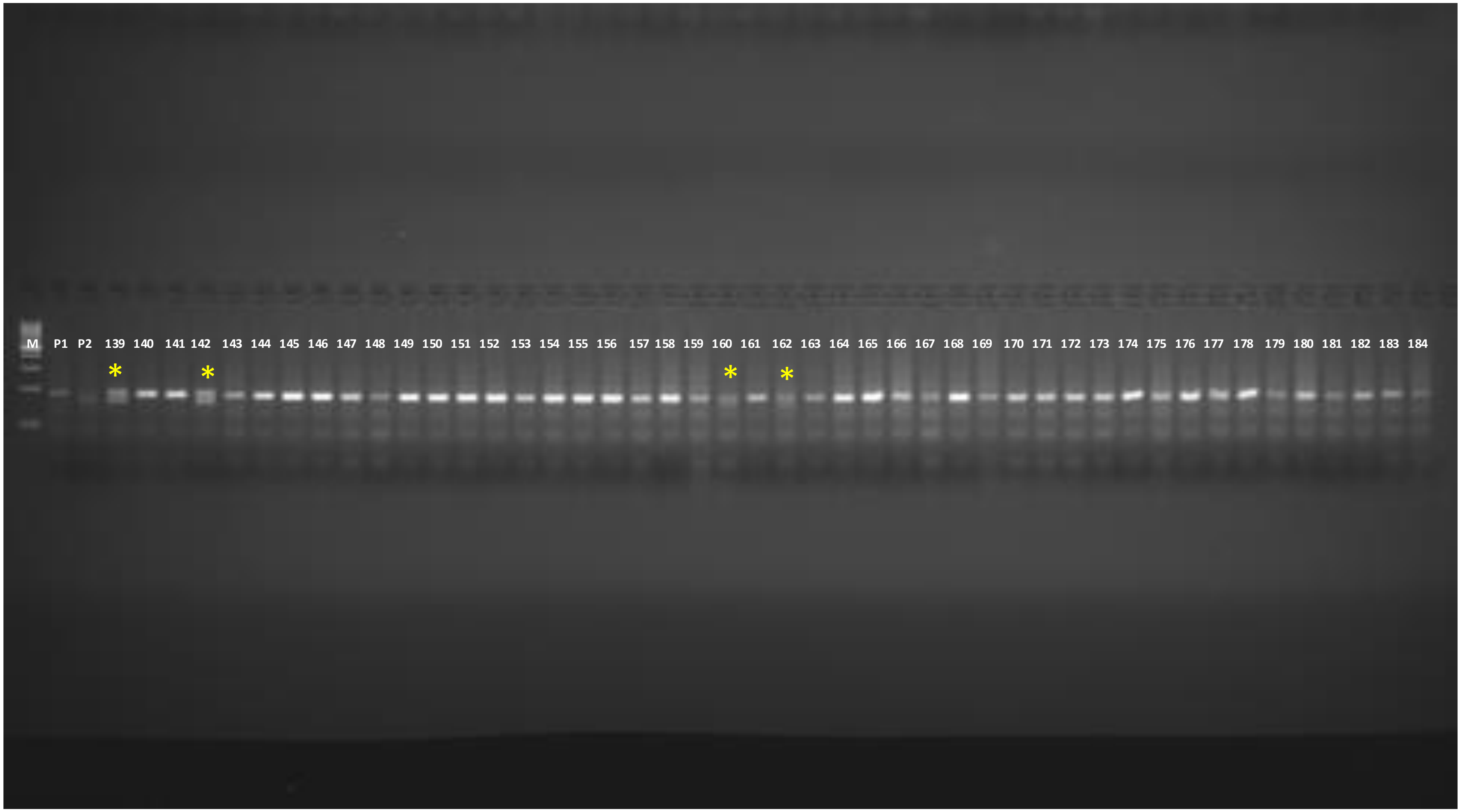
M P1 P2 139 140 141 142 143 144 145 146 147 148 149 150 151 152 153 154 155 156 157 158 159 160 161 162 163 164 165 166 167 168 169 170 171 172 173 174 175 176 177 178 179 180 181 182 183 184
* * * *

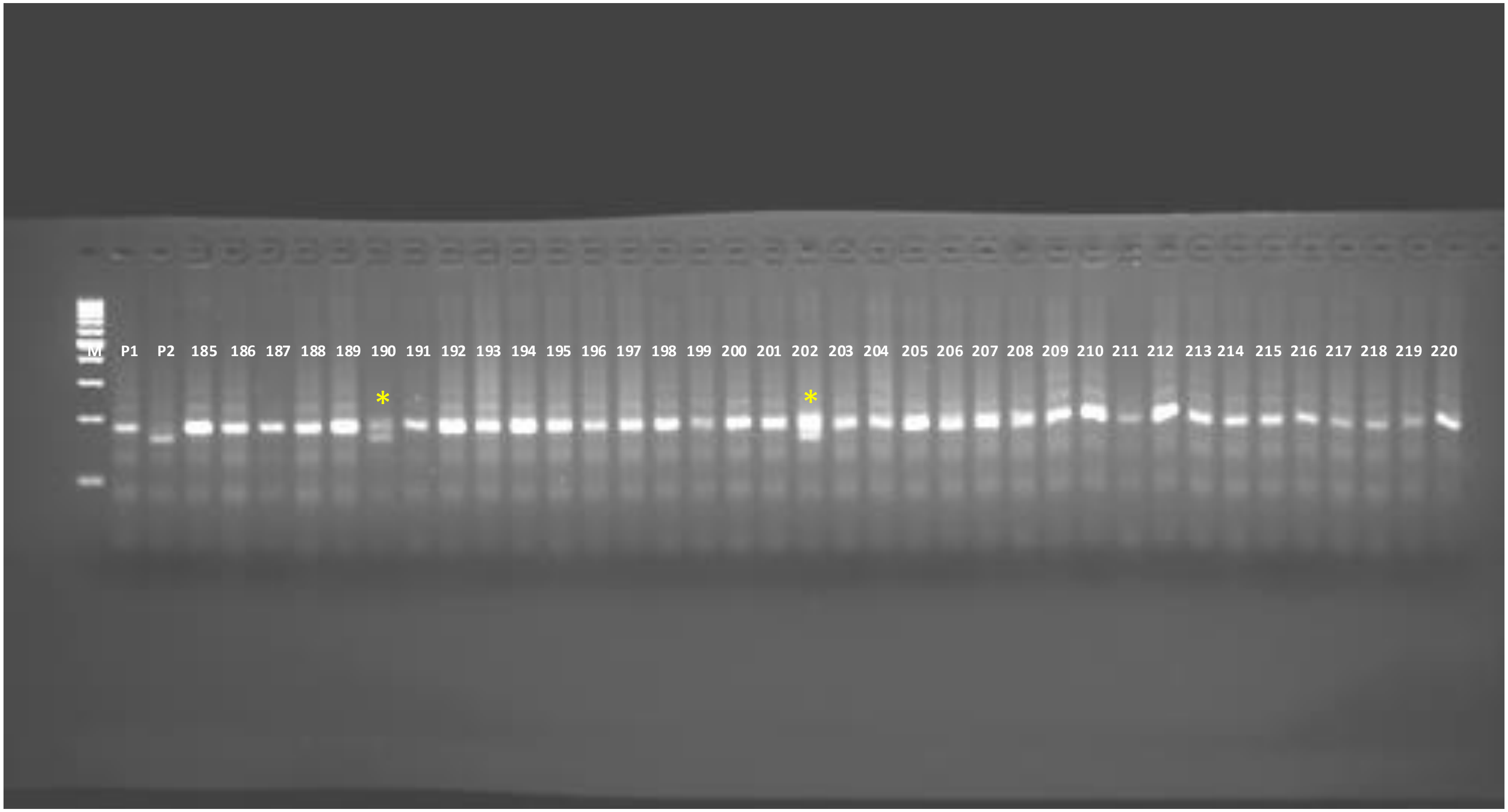
M P1 P2 185 186 187 188 189 190 191 192 193 194 195 196 197 198 199 200 201 202 203 204 205 206 207 208 209 210 211 212 213 214 215 216 217 218 219 220

Supplementary Figure S2. Genotyping of the recombinants detected with STS-5 and RM1261 with internal polymorphic markers. a) Genotyping of recombinants detected with RM1261 with RRS77. b) Genotyping of recombinants detected with RM1261 with RRS16. c) Genotyping of recombinants detected with STS-5 and RM1261 with RRS12. P1= HPU2216; P2= RIL4.

a) Genotyping of recombinants detected with RM1261 with RRS77

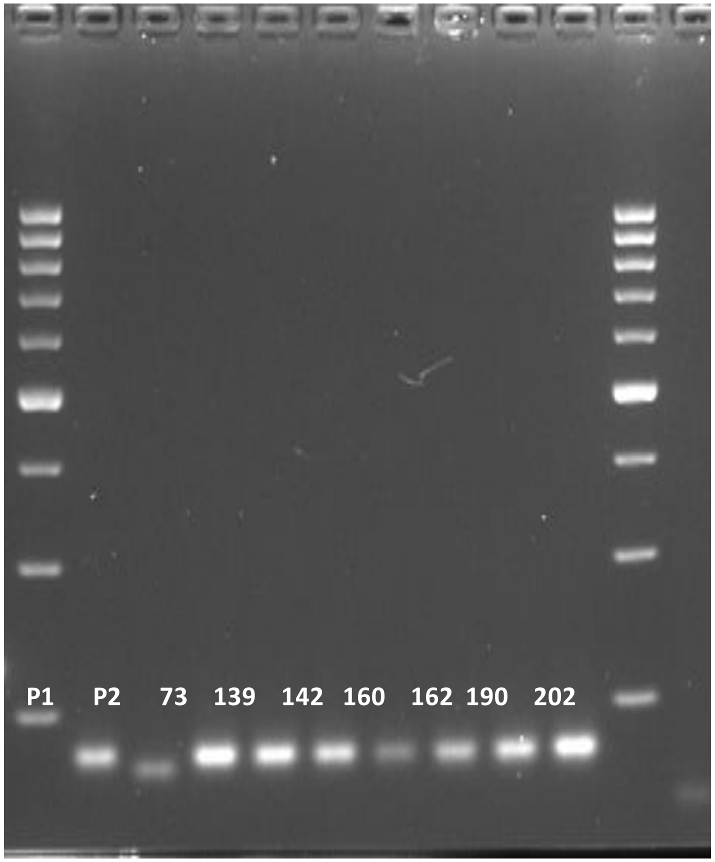

b) Genotyping of recombinants detected with RM1261 with RRS16.

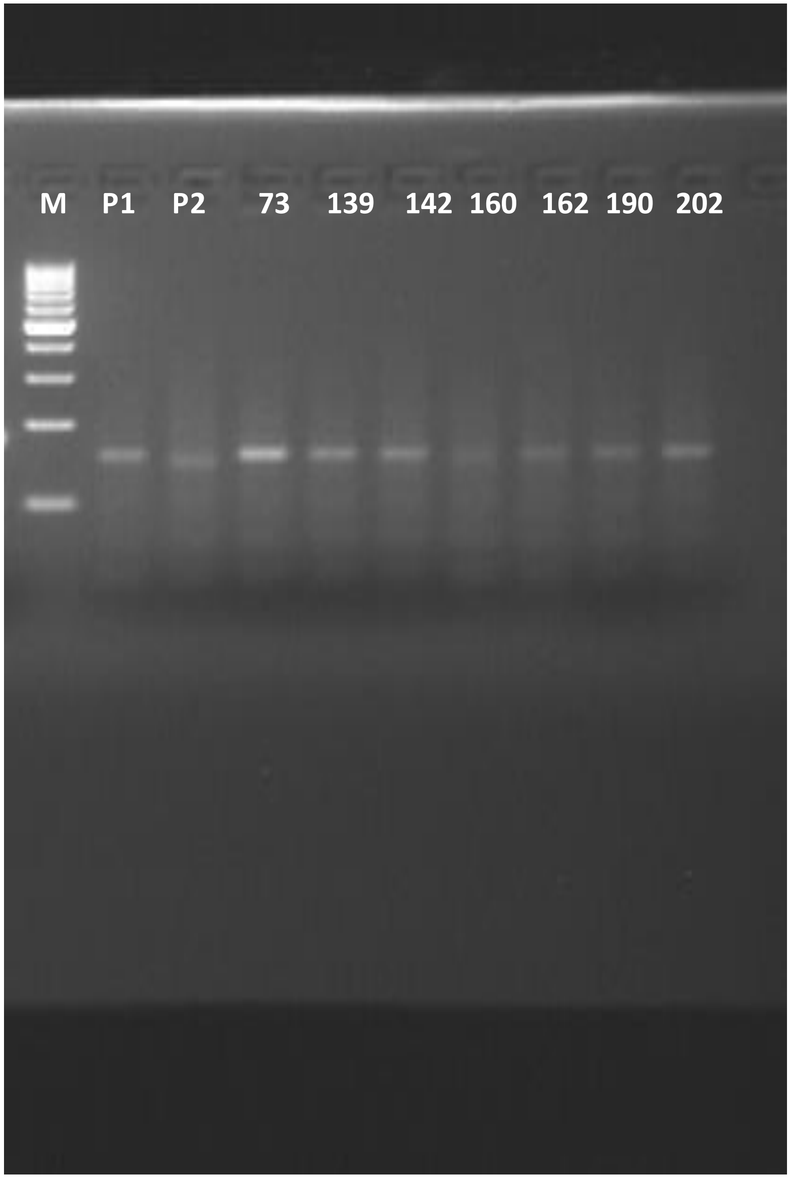

c) Genotyping of recombinants detected with STS-5 and RM1261 with RRS12.

M P1 P2 63 69 74 77 86 124 158 181 185 193 73 139 14 2 160 162 190 202

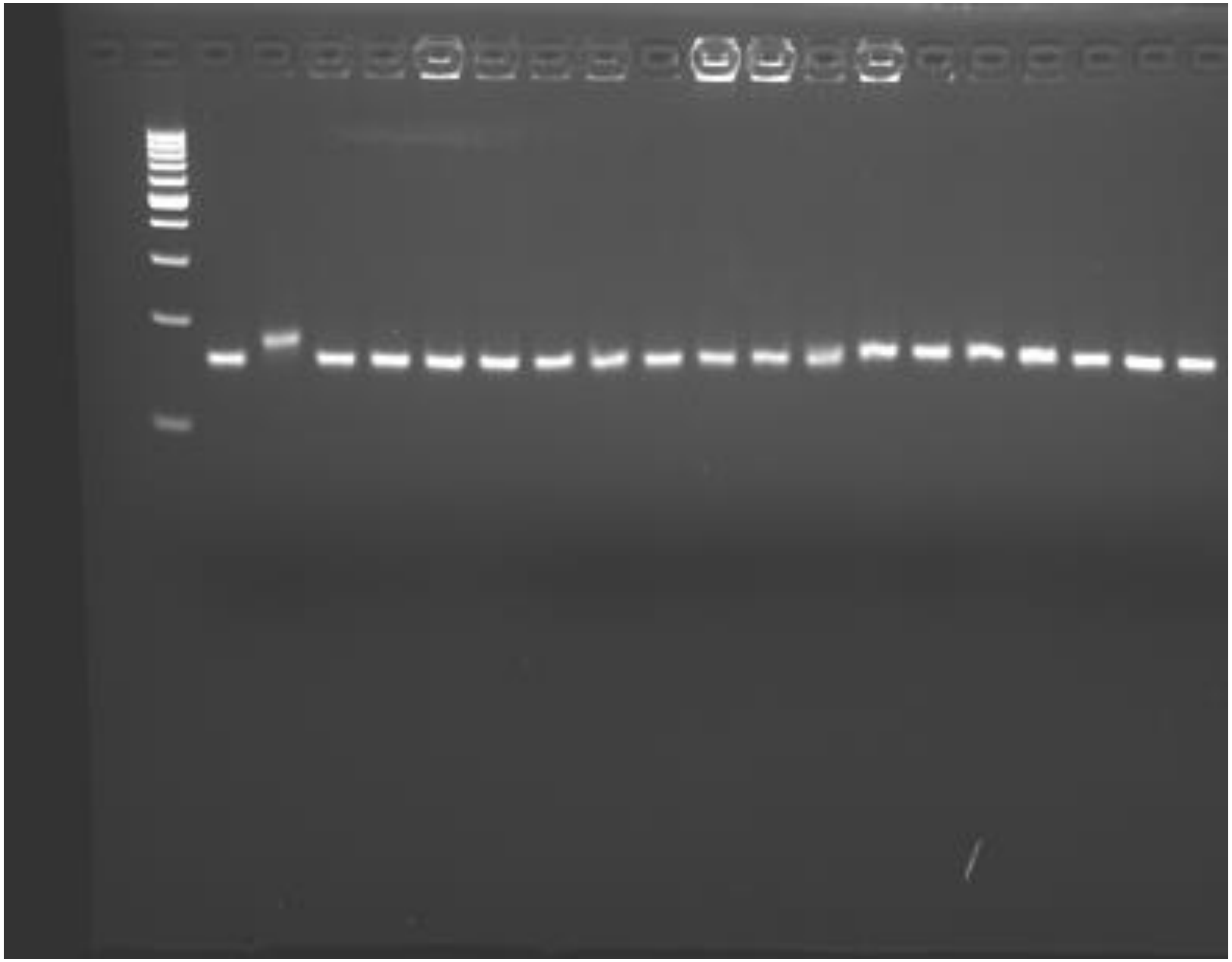

Supplement: Supplementary file 1 — Supplementary Information. [file 41598_2023_46070_MOESM1_ESM.pdf]
